# Explaining the Green Development Behavior of Local Governments for Sustainable Development: Evidence from China

**DOI:** 10.3390/bs13100813

**Published:** 2023-10-01

**Authors:** Jianguo Du, Xiaowen Zhu, Xingwei Li, Enes Ünal, Philip Longhurst

**Affiliations:** 1 School of Management, Jiangsu University, Zhenjiang 212013, Chinaxiaowen.zhu@cranfield.ac.uk (X.Z.); 2 Centre for Design Engineering, Cranfield University, College Road, Cranfield MK43 0AL, UK; e.unal@cranfield.ac.uk; 3 College of Architecture and Urban-Rural Planning, Sichuan Agricultural University, Chengdu 611830, China; 4 School of Water, Energy and Environment, Cranfield University, Cranfield MK43 0AL, UK; p.j.longhurst@cranfield.ac.uk

**Keywords:** local government, green development behavior, new institutionalism, organizational process research, grounded theory

## Abstract

Although researchers have examined organizational sustainability practices, a specific interpretation of local government green development practices remains for supplemental analysis. This study conducted an empirical survey of 53 local officials from departments related to green development to understand the key processes and practices of green development behavior of local governments in China. The key findings indicate that the main stakeholders involved in the green development practices of Chinese local governments consist of enterprises and residents. In part, local government green development practices emphasize the greening of enterprises, especially in the step of process environmental regulation. The new institutionalism theory and the organizational process research provide dependable insights into green development behaviors. Our findings further shed light on the process of cross-sectoral cooperation across local government departments in green development, contributing to local multi-sectoral interactions for regional green development.

## 1. Introduction

The 2030 Agenda for Sustainable Development and the Sustainable Development Goals emphasize the importance of local stakeholders. Local governments are in an important position to implement localized intermediary strategies for the global agenda [1]. With the increasing contradiction between emission reduction and economic growth [2,3], the carbon emission situation remains gloomy. In 2022, global energy-related CO2 emissions increased by 0.9%. As an essential tool for climate mitigation, green development has attracted significant attention from developed countries [4,5]. Since the beginning of the 21st century, the United Nations has issued the “China Human Development Report 2002: green development” [6]. The theoretical frameworks and practices of green development have become indispensable elements for Chinese government policymakers to discuss policies [7]. Notably, Chinese policymakers have contributed to the practice of reducing emissions and promoting clean energy [8,9,10]. However, following a report released by the United Nations Environment Program in 2021, experts predict that governments participating in the 2030 Paris Agreement will miss their carbon reduction targets [3]. Implementing green development behaviors is a crucial government initiative to reverse this situation [1,3]. Therefore, to encourage the growth of green production modes for businesses and green lifestyles for the general public, it is vital to investigate municipal governments’ behavior in green development.

Local governments play a crucial role in green development [1]. On the one hand, local governments have the authority to enact regulations guiding businesses and citizens toward environmentally responsible conduct [11]. On the other hand, local governments have an advantage in obtaining local information, allowing them to provide public goods better suited to residents’ preferences than the central governments [12]. Consequently, local governments contribute to a more efficient implementation of national policies [13]. However, the main problem local governments in China currently face in implementing green development practices includes the lack of incentives to implement effective environmental governance measures according to local situations [14]. Second, because of the high threshold of green development and the difficulty of implementation [15], local governments still encounter the problem of selective implementation [16]. Moreover, the traditional localized and disorganized model of local governments as key players in environmental governance reduces governance efficiency [17]. Accordingly, localizing green development practices necessitates research to understand the fundamental practices and processes for implementation.

Existing research on the implementation of green development practices by local governments covers different aspects of policy and public action, including planning for emission reduction targets [18,19], selecting green policy instruments [16,20], behavioral changes in local governments [21], and implementing public green procurement [22,23]. Research on implementing green development practices by local governments in China mainly focuses on environmental regulation [24,25,26,27,28], decision making on green development behaviors [3,15,17,29], and green behavior of civil servants [30].

Accordingly, this study proposes the main research question: what does the green development behavior of local governments comprise? This study interviewed 53 local government officials using local governments in China as a case study to answer this question. Local governments’ green development endeavors are usually associated with the Development and Reform Commission, the Ecological Environment Bureau, and the Water Bureau [20]. These departments are mainly responsible for economic, environmental, social, scientific, and technological development. This study aims to analyze the composition of local governments’ green development behaviors and critical processes and practices of its implementation by combining the new institutionalism theory with organizational process research. Hence, it can better expand the localization research on green sustainable development practices.

Compared to previous research, our study has made the following contributions. First, new institutionalist theory and organizational process research have similar characteristics that help explain organizational change [12,31,32,33]. Therefore, this study deconstructs the idea that local governments’ green development behaviors consist of two aspects, the formulation and implementation of green development policies, and analyzes the key processes and practices of local governments’ green development behaviors. Second, this study contributes to understanding the green development behavior of local governments in China. Most studies have been conducted on the European [21,34] and American [18,35] regions, while Chinese studies have received less attention. The process of local governments in China to localize green, sustainable practices is not clear. Furthermore, the findings detail the measures taken by local governments to promote the greening of business production methods and public lifestyles. Significantly, local governments in China have paid more attention to corporate green production than to greening the public’s life. However, existing studies have emphasized the relationship between communities, residents, and local governments’ sustainable practices [35]. Nevertheless, the partnership between local governments and the private sector should not be ignored [10]. Our research results not only help local government departments and stakeholders to deepen their understanding of green development but also help local governments to realize regional green development.

This research is organized as follows. The second section examines the relevant research on local government in green development and the pertinent theories underlying the practice of local government green development. The third section explains the research design, data collection, and analysis. In the fourth section, we analyze the research findings. In the fifth section, this study summarizes and discusses the research findings in greater detail. Finally, this study’s conclusions, implications, and limitations are outlined.

## 2. Theoretical Background

### 2.1. The Role of Local Governments in Green Development

Local governments, as critical actors in green development practices, influence many key emission sectors, including buildings, energy supply, transportation, planning, and waste management [21]. Meanwhile, local governments serve as facilitators and leaders in guiding the sustainable transformation of business and society [36]. Notably, local governments adopt different rhythms when implementing green sustainability policies and practices depending on the country, administrative level (for example, local versus central), or the activities and goals of each organization [34]. Therefore, academics do not have a unified definition of local government green development behaviors.

Some scholars have linked green development behaviors to plans, programs, and goals. Wheeler noted that state and local governments in the US typically implement sustainable practices via planning and that most projects have set emission reduction targets, created emission inventories, and greened public sector operations [18]. Deslatte and Swann (2016) examined cities’ choices of green policy tools regarding organizational goals, such as greenhouse gas emission reductions and energy efficiency [16]. Additionally, local governments often seek environmental goals that exceed state and federal minimum requirements [20]. Local governments have adopted comprehensive plans emphasizing sustainability in local sustainability and climate action plans [19].

Other scholars perceived green development behavior as a behavioral change. Revell believed that behavioral change is a policy tool and behavioral change has been widely developed in environmental policy to encourage sustainable lifestyles [21]. Accordingly, this article identifies local government green development behavior as a change in behavior, namely behavior taken by the local government to change from the original development mode of pursuing only economic growth to the green development mode of seeking environmental protection and economic growth. The purpose is to encourage enterprises to realize the greening of production methods and residents to learn the greening of lifestyles.

Furthermore, as the concept of green development emerged, local governments’ key role in the green development process in China has gradually emerged (as shown in Table 1). Studies on local governments in China mainly focus on the regional level and are generally presented using panel data and empirical analysis [24,25,26,27,28]. Moreover, other scholars researched the behavior of local governments in green development and the green behavior of civil servants with structural equation modeling, evolutionary games, and meta-analysis methods [3,17,29,30]. Huang et al. investigated collaborative behavior in local government water governance using questionnaires and semi-structured interviews [17]. Regarding the following questions, which theoretical frameworks can be used for analysis remains to be studied: What does the green development behavior of local governments comprise? What are the key processes and practices of green development behavior? Accordingly, this research presents case studies of Chinese local governments combining the institutional theory and the organizational process theory to provide a specific analysis of green development behaviors.

### 2.2. Theoretical Analysis of the Green Development Behavior of Local Governments

Institutional theory has been applied to studying organizational responsiveness to environmental issues [31]. It is frequently used to describe how new practices are adopted in organizations [32]. Institutional theory offers explorations of different methods/mechanisms through which information about legitimate and socially acceptable organizational behaviors can be conveyed, and such behaviors can be institutionalized in organizations [18]. Therefore, one of the goals of our study is to apply institutional theory to explain the process of institutionalizing green development practices in local governments.

On the one hand, the new institutionalism theory highlights three conceptually distinct mechanisms by which organizations reflect their institutional environment: mandatory, imitative, and normative [31]. Do local governments’ green development behaviors involve these three essential characteristics? To address this targeted question, this study applies rational choice institutionalism and social institutionalism as analytical tools. These two institutionalisms can be used as analytical tools to explore the institutionalization of sustainable development in local governments [39]. Dunlop and Russel analyzed the role of UK regulators in integrating sustainable development into public services based on rational choice and sociological institutionalism [40]. Page pointed out that governance studies can generally be categorized into sensible choice and sociological institutionalism [41]. Andrews-Speed viewed the energy sector as a socio-technical system, drawing on the core ideas of rational choice and socio-organizational institutionalism to explain the socio-technical transformation in depth [42].

On the other hand, new institutionalism theory is often criticized for only explaining persistence and homogeneity, but recent studies have shown its suitability for explaining change [12]. Local governments’ green development behaviors reflect dynamic changes in behavior. Process research in organizational theory suggests that organizations cannot simply replicate the practical processes of other firms [33]. Langley and Tsoukas note that a particular organizational process is usually adequate, but nothing is known about how it is achieved [43]. Therefore, it is necessary to gain insight into the sequence of events over time [44]. This evolutionary perspective explains how and why a process unfolds over time [45]. This study describes local governments’ essential processes and practices in implementing green development behaviors at different times and in the face of different stakeholders, explained from an evolutionary perspective.

## 3. Method

### 3.1. Research Design

This article employs the case study method [46]. The case study method applies to new phenomena that must be explored and serendipitous discoveries in exceptional circumstances [47]. In this study, the specific processes implemented by local organizations in transforming local government behaviors to green development behaviors are yet to be explored, so an in-depth case study methodology was adopted to understand the phenomenon comprehensively. An in-depth case study analysis is consistent with the objectives of this paper for the following reasons. First, the leadership of local officials as decision makers is dynamic, multifaceted, and complex. It manifests itself in context [48], which requires an in-depth look at how these processes evolve in sequences and transitions [36]. Second, multiple case studies have generated rich, field-based insights into the critical strategies by which local governments develop green development policies and implement green development practices.

### 3.2. Data Collection

This study focuses on the local government departments in Jiangsu Province responsible for green development. Jiangsu Province is in the eastern coastal region of China and ranks as the second largest province economically. Since this study focuses on local governments, the local level is defined as local government units with different population sizes, municipalities (such as cities, towns, and villages), and district-level governments [10]. We selected three prefecture-level cities in Jiangsu province in the southern and central regions (detailed sample information is shown in Table 2). The target group of our study is the staff of local government departments related to green development. While the job titles of the respondents varied slightly across the region, local green development efforts were typically associated with nine departments, including the Development and Reform Commission, the Bureau of Ecology and Environment, and the Water Authority [20]. All participants in our study view green development, environmental protection, low-carbon circular development, and cross-sector cooperation as integral to their work. The research data consists of 53 semi-structured interviews with local government officials, ranging in duration from 0.5 to 1.5 h for each interview. Finally, a memorandum of approximately 304,000 words was composed. Additionally, 23 archival data were collected, including policy documents related to green development practices issued by local governments. Each interview will be transcribed and organized using NVivo12 (Lumivero, Denver, CO, USA). Table 3 contains detailed information about the respondents.

In addition, the organization sample includes nine local government departments, including the Development and Reform Commission, Bureau of Industry and Information Technology, Bureau of Ecology and Environment, Bureau of Science and Technology, Bureau of City Administration, Bureau of Agriculture and Rural Affairs, Bureau of Commerce, Water Authority, and the Bureau of Housing and Urban-Rural Development. Notably, the Development and Reform Commission and the Water Authority have at most eight respondents. The results section will specifically elaborate on the roles of these sectors in the process of green development. In general, our sample reflects not only the differences in the functions of local government departments but also, to a certain extent, the importance of cross-sectoral cooperation. To further improve the accuracy and dependability of this study, the authors all participated in a discussion regarding data analysis and interview coding verification.

The in-depth interviews were conducted one-on-one without the interference of other people to enhance the integrity and efficiency of data collection. All interviews were conducted and recorded in Chinese, which is the mother tongue of the prominent researchers and participants. It is helpful for interviewers to quickly establish a harmonious relationship with respondents and obtain rich data [49]. At the same time, before recording the interview, participants were asked to read a brief description, including detailed information such as the purpose of the survey and the confidentiality of the results [50]. To further ensure the privacy and confidentiality of participants, we anonymously numbered the respondents during the verbatim transcription of the interview [49].

### 3.3. Data Analysis

**Figure 1:**
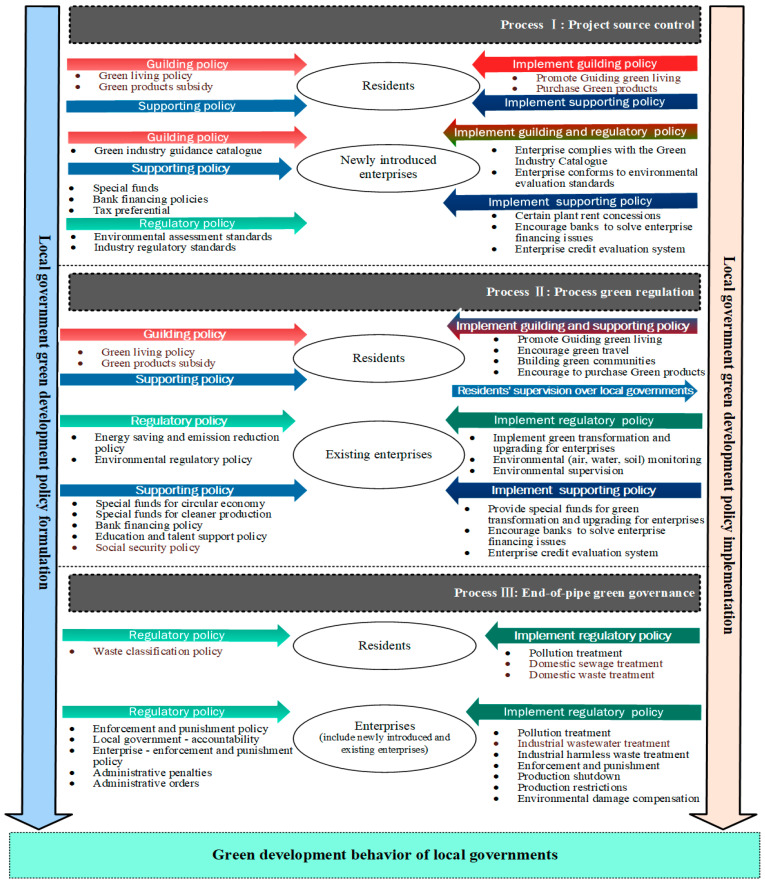
The conceptual model of green development behavior of local governments.

The traditional coding process for content analysis [51] relies heavily on analyzing and interpreting the data [52]. Specifically, the axial coding process involves examining the categories constructed from the subcategories and the possible relationships between them based on the data [52,53]. Therefore, this study analyzes the specific attributes of the categories or phenomena generated (mandatory, incentivized, and normative properties of local governments’ green development behaviors) and critical processes for implementing green development behaviors. Accordingly, the theoretical framework of this study provides a clear roadmap for processing the coding and translating the results into Figure 1.

Additionally, this study strictly followed the steps recommended by Tellis [54] to triangulate the information collected from both primary and secondary sources. First, each author independently reviewed all the information from the transcribed interviews and secondary documents to verify their validity and avoid potentially ambiguous and equivocal data to be included in the database. Then, each author compared or corroborated their own analyses with the ones of other authors to reach a shared understanding and interpretation of the whole information under investigation. Finally, the authors triangulated all the information received [55]. This research examined the framework to create conceptual labels, categories, and subcategories for any given time.

## 4. Results

This section describes the qualitative research findings that led to the core category of “local government green development behavior”. Table 4 displays the overview of the key processes and practices for implementing green development behaviors by local governments. Our findings indicate that local government green development behavior consists of formulating and implementing green development policies. In the following section, we will elaborate on these two categories.

### 4.1. Green Development Policy Formulation of Local Governments

Local government green development policy formulation is, in fact, the beginning of the implementation of green development behaviors. Process research in organizational theory suggests the need to understand more about the emergence of organizational processes [33]. Clarifying the localized green development policies formulated by local governments is a prerequisite to accurately implementing green development behaviors. According to rational choice institutionalism, acting as an agent, namely an implementer, of the central government’s implementation of green development policies [40], local governments usually seek to maximize their own utility. This means that following the green development policies proposed by the central government, local governments develop green development policies in accordance with local circumstances. Alternatively, sociological institutionalism emphasizes that regulators act according to the way they perceive to be appropriate for their roles and particular policy sectors [40]. Accordingly, this paper focuses on analyzing three core areas of local governments’ green development policymaking: green development guiding policies, supporting policies, and regulatory policies.

#### 4.1.1. Green Development Guiding Policy Formulation

Green development guiding policies include enterprise green production and public green life-guiding policies. The complexity of the local government and its institutional environment necessitates that when formulating green development policies, local governments consider the stakeholders’ interests. In our research, we regard the green development guiding policy as a binding norm, reflecting the preferences of members of closely related groups. Second, norms are usually regarded as industry associations’ values and standards of conduct [56]. Standardized norms not only help local government officials better understand their roles but also help officials clearly understand the job responsibilities of other colleagues [57]. Therefore, as conceptualized in this study, the green development guidance policies formulated by local governments include the green production policy for guiding enterprises and the green life policy for guiding the public.

- In guiding the green development of local industries, normativity is regarded as the main priority. It is believed that normative pressure can influence the environmental practices of businesses [56]. Our data indicate that local governments’ green industry guidance catalog is the most crucial policy for directing the green production of industries. This policy is the green industry guidance catalog proposed by the central government in 2018 [24], formulated by local governments to guide the green development of industries following actual local conditions. We interviewed local government departments involved in green development, and a consensus emerged that “the green industry guidance directory had become a critical necessity for local governments to introduce businesses” (Development and Reform Commission, P2). Second, it must be emphasized that the Development and Reform Commission and the Industry and Information Technology Departments of local governments are the two primary departments responsible for formulating green industry guidance policies.

The following is the description of an interviewee from the Department of the Development and Reform Commission: “In terms of industry selection, the industries encouraged to develop in the documents issued by our city include new materials and new energy. These industries are developed according to the green industry guidance catalog formulated by the Development and Reform Commission and in combination with the actual local characteristics of our city”.

- In addition to developing policies to direct the green development of industries, the public is another stakeholder in the green development of local governments. Respondents from the Bureau of Ecology and Environment stated that domestic sewage discharge, food waste, garbage classification, and other public behaviors forced local governments to confront this long-term environmental problem in daily life. Nonetheless, the key to addressing this issue is promoting the greening of the public lifestyle. Citizens and consumers can alter their lifestyles or exert pressure on the government and businesses [11]. In 2020, the central government of China issued top-level guidance on building a modern environmental governance system, pointing out that promoting a “green lifestyle” is regarded as one of the essential tasks for China’s green development [58]. Our data also demonstrate that local governments have actively formulated subsidy policies for purchasing green products, preferential policies for green travel, and innovative green development concepts such as the “green campus and green community” (Development and Reform Commission, P17). Additionally, for rural residents, officials from the Bureau of Agriculture and Rural Affairs stated the following:

“*Our department actively promotes soil testing and formula fertilization technology, green prevention and control technology of crop diseases and pests and guiding rural residents to use biological pesticides actively*”

(Bureau of Agriculture and Rural Affairs, P10).

#### 4.1.2. Green Development Supporting Policy Formulation

Rational choice institutionalism assumes that policies are not neutral but have different incentives for different behaviors [59]. Compared to obligatory policies, incentive policies can offer more adaptable economic incentives, making them more efficient. Gao believed that the market expansion caused by incentive policies contributes to improving normative and cognitive legitimacy in society [60]. Our research also indicates that “*many local governments are utilizing market-based solutions to address the issue of enterprise incentives*” (Development and Reform Commission, P17). Because of this information, we will introduce the green development support policies formulated by local governments, such as financial incentive policies, education and talent policies, and social security policies.

- The fiscal incentive policy is intended to provide funding and tax incentives to businesses that meet green standards. For instance, “*Provide special funds for businesses to conserve energy and develop a circular economy*” and “*encourage banks and other financial institutions to increase business financing*”. This section primarily divides fiscal incentive policies into investment and financing policies and preferential tax policies. These financial incentive policies rely on the collective efforts of normative actors, including local governments, businesses, and industry associations [52]. Fiscal incentives can effectively increase the market size of green industries such as the solar energy industry. Additionally, local governments may develop preferential tax policies for businesses that meet green standards following applicable national tax policies. A representative of the Department of Industry and Information Technology confirmed this.

“*Our district and even Industrial Park level will grow their green industry incentive policies, mainly financial incentives for green factories and supply chains. For instance, the incentive policies that support the growth of the renewable resource recovery industry can take the form of tax-exempt preferential policies and applications for special funds when the investment reaches a certain threshold. Second, certain regions have relevant incentive policies for cleaner production. Reward companies or projects that achieve cleaner production, for instance, to raise the enthusiasm of enterprises in this region for cleaner production*” (Bureau of Industry and Information Technology, P16). In addition, it is crucial to highlight that due to the heterogeneity of the industry, various local government departments will implement green development incentive policies based on the actual situation of each industry and even individual businesses, making the formulation of these policies flexible.

- By providing education and talent support, enterprises can promote green development and form an excellent social demonstration effect. Our interview data indicate that local governments have formulated education and talent-related policies. In addition, it is necessary to emphasize the transformation of local government functions and the significance of active service. Specifically, “*Local authorities will answer some of the environmental policy questions raised by companies at their environmental reception days*” (Bureau of Ecology and Environment, P18). “*When businesses encounter pollution issues, our office will take the initiative to assist them in securing the technical assistance they need to actively resolve their pollution issues*” (Bureau of Science and Technology, P52).
- The social security policies by local governments for businesses cannot be overlooked in our survey results. Specifically, during the transitional phase of green development, local governments implement social security policies that facilitate the transformation and upgrading of businesses. The informant of the Department of Industry and Information Technology stated, “During the transition period of green development, businesses may face challenges. The local government has, therefore, provided them with social security policies to assist businesses in overcoming obstacles. In addition, the local government will collaborate with the neighborhood office to assist businesses in resolving issues such as re-employment and labor security for vocational workers” (Bureau of Industry and Information Technology, P16).

#### 4.1.3. Green Development Regulatory Policy Formulation

The regulatory policy generally refers to encouraging or restricting activities or actors through legislation, monitoring, and sanctions [61]. On the one hand, government policies can provide the legal framework for businesses to enter a region, such as environmental protection and energy consumption evaluation standards, and introduce green industries from the ground up to promote local green development. On the other hand, the obligatory pressure exerted by entities in the institutional environment on the organization restricts its strategic options. It forces it to meet institutional expectations, such as enhancing regulatory review and monitoring to promote green transformation and upgrade regional enterprises. We categorize local government green development regulatory policies as energy conservation and emission reduction policies, environmental regulation policies, and law enforcement and punishment policies.

- Energy conservation and emission reduction policies refer to specific, detailed green goals with measurable indicators. By analyzing the survey data, we determined that local government departments have reached a consensus on the importance of energy conservation and emission reduction policies in promoting regional green development. On the one hand, the roles of energy conservation and emission reduction policies vary across departments. For instance, local Development and Reform Commission departments will establish energy conservation and emission reduction goals annually for the region. On the other hand, the Water Authority’s policy regarding energy conservation and emission reduction is “*water conservation and emission reduction transformation in high water consumption industries, improving the recycling rate of industrial water, and promoting water conservation and pollution reduction*” (Water Authority, P42). The Bureau of Housing and Urban-Rural Development’s energy conservation and emission reduction policies are expressed as “*Water, material, land, and energy conservation. Water conservation refers specifically to water conservation and rainwater recycling. Wood and other materials can be conserved using prefabricated buildings. The plot ratio primarily reflects land conservation, which helps to conserve land. Finally, energy conservation, including using renewable energy sources such as solar energy, ground source heat pumps, and photovoltaic power generation*”.
- Local governments seem to be the direct legal subordinate institutions of the central government. The government agency responsible for formulating environmental regulations is the Environmental and Ecological Bureau [62]. The pursuit of legitimacy is the key driving force for enterprises to decide on environmental response behavior. It is not difficult to see that the formulation of environmental regulatory policies has a restrictive or incentivized impact on the green development of enterprises. According to our data analysis, environmental regulatory policies include strict environmental regulations and environmental assessment standards. For example, “*the environmental rules of the water conservancy department include the most stringent water resources management system and the ecological red line policy issued by the state*” (Water Authority, P43). According to national environmental regulations, the provincial and municipal levels also formulate related environmental supervision policies or regional standards, such as the provincial “263 plan” (Bureau of Housing and Urban-Rural Development, P20), “*environmental governance of land pollution*” (Bureau of Agriculture and Rural Affairs, p23), municipal “*water source protection regulations*” (Water Authority, P44), and “*Taihu Lake standard*” (Bureau of Housing and Urban-Rural Development, P48). The environmental assessment standard here is an essential means to ensure that the regional environmental indicators meet the criteria, and this consensus has been certified by many departments. Respondents from the Bureau of Ecology and Environment advised that “*when introducing projects, all projects must pass the environmental assessment, and projects that do not meet the environmental assessment standards cannot enter the region*” (Bureau of Ecology and Environment, P35). In addition, it controls “*total energy consumption*” (Development and Reform Commission, P17). It is not difficult to see that the routine activities of local governments are inseparable from environmental regulatory policies, which are the core components of green development regulatory policies.
- Law enforcement and punishment policies should also be implemented to ensure the implementation of the green development policy and realize the region’s green development. Our findings indicate that law enforcement policies include administrative punishment and order, such as “*production limits, administrative penalties, and time-limited disciplinary measures. The most severe administrative penalty provided for by the law is the suspension of production for remedial purposes*” (Bureau of Ecology and Environment, P32). Since the implementation of the newly revised environmental protection law in 2015, the specific rules involved in each case of polluting businesses have varied to varying degrees, according to the head of the ecological environment division. For example, “*there is a daily method for determining the severity of punishment; the environmental law enforcement department notifies the business of its violations, which it must fix immediately or face a fine if the deadline is not met*” (Bureau of Ecology and Environment, P18).

### 4.2. Green Development Policy Implementation of Local Governments

Policy implementation requires government actors to manage and implement the policy [41], including various actions, interpretation, dissemination, experimentation, coordination, and control. Although the above studies describe green development policymaking, policy formulation to implementation would probably not be easy. First, implementing development behaviors is a dynamic process that denotes the transformation of policy content into practical effects to achieve the stated goals of the policy. It also means moving from the previous model of pursuing only economic growth to the model of pursuing green development. Second, process research in organizational theory suggests that organizations cannot simply replicate processes that work effectively in other organizations [33]. Understanding how processes are achieved requires insights into the sequence of events that occur over time [43]. The third, rational choice, institutionalism, proposes that different green development behaviors should be implemented according to the key stakeholders in the process of implementing green development behaviors [41]. Finally, since implementing green development behaviors in local governments involves the assistance of multiple departments, local government officials usually act upon what they believe is appropriate for their roles and specific policy sectors [40]. Therefore, this section highlights the key processes and practices of local government departments in implementing green development behaviors when interacting with the public and businesses, including source project control, process green regulation, and end-of-pipe green governance.

#### 4.2.1. Project Source Control

Project source control is a prerequisite for local governments to implement green development behaviors, which includes publicizing green development policies, local governments guiding enterprises to green production and the public to green living, and describing the access standards for new enterprises. According to sociological institutionalism, norms not only “bind” but also “shape” or “constitute” behavior [63]. Individual preference formation among actors is an endogenous process influenced by informal systems such as norms and beliefs. Therefore, guiding policies can be viewed as obligatory normative policies, which help increase enterprise and public awareness and understanding of green development.

- Local governments’ publicity of green development policy is an essential prerequisite for effective policy implementation. Zhang et al. stated that businesses should fully comprehend the government’s green-related policies, which will help them establish a green concept [64]. Development and Reform Commission respondents stated that “we could disseminate industrial policies to assist businesses in better understanding these policies” (Development and Reform Commission, P36). Furthermore, he mentioned, “*At present, there are many channels of publicity, such as social media, television, newspapers, NGOs*”. Meanwhile, this informant also stressed that local governments could take the initiative to provide services, which will help local governments fully understand the current situation of enterprises. Such two-way interaction helps realize information sharing between local governments and enterprises, thus helping to improve the implementation effect of policies. In addition to promoting green development policies for enterprises, local governments also provide relevant policy publicity for the public, such as “*publicity of waste classification*” and “*publicity of green ecological breeding mode and technology*”.
- The green development guiding policy implementation also includes enterprises and the public. Local governments typically guide and encourage voluntary green production by green industrial policies (such as the Green Industry Guidance Catalog) and phase out obsolete and highly polluting equipment in terms of technological transformation, as opposed to interfering with the normal development of businesses (Bureau of Industry and Information Technology, P36). Participants from the ecological and environmental sector asserted that “*government departments should avoid a one-size-fits-all approach and instead guide and encourage enterprises with new ways of starting a business or altering their original production methods*” (Bureau of Ecology and Environment, P32). Additionally, to further promote the greening of public lifestyles, the City Administration department has also established “*particular actions for waste classification and treatment and developed facility and operation management standards*” (Bureau of City Administration, P7).
- Project source control means that when introducing new enterprise projects, local governments must comply with the relevant provisions of the green industrial policy. Then, they comply with the environmental assessment standards to prevent enterprises with high pollution and high emissions from entering the region from the source. Before introducing the enterprises, the Bureau of Commerce will hold a pretrial meeting, equivalent to a cross-departmental consultation mechanism. Then, all functional departments, including the Bureau of Ecology and Environment, Development and Reform Commission, will approve the project.

Specifically, “*The development and reform department is mainly responsible for approving projects in this part. First, it is necessary to ensure that new projects have low energy consumption. Second, the environmental assessment standard is also an important indicator in project approval*” (Development and Reform Department, P33). This notion is consistent with the Industry and Information Technology Departments. The interviewees of this department stated, “*Currently, the primary industries to be introduced are the new materials industry and the Internet of Things industry, and only businesses that meet environmental assessment criteria can be introduced. This way, enterprises with high pollution and high emissions can be controlled at the source*” (Bureau of Industry and Information Technology, P16).

- Incentive policies affect different enterprises, so local governments need to implement differentiated incentive policies. The impact of financing incentives on the formation of new companies is different between start-ups and new subsidiaries [60]. Our research results confirm that local governments have other incentive policies for newly introduced and existing enterprises. As suggested by the participant, “*For newly introduced enterprises, they must first meet the requirements of the green industry, and they enjoy certain plant rent concessions. Second, local governments will give different incentive policies in enterprise incubation and industrial transformation stages. Third, local governments will encourage banks and other financial institutions to resolve enterprise financing issues*” (Development and Reform Commission, P38). However, it is necessary to emphasize “*The enterprise credit evaluation system, which divides enterprises into five colors: red, yellow, blue, green and black. Financial institutions will provide financial credit by dividing colors to promote enterprises’ green transformation and upgrading*” (Bureau of Ecology and Environment, P31).

#### 4.2.2. Process Green Regulation

Process green regulation is central to effectively implementing green development behaviors by local governments. Due to the complexity and unpredictability of local governments and their institutional environments, it is necessary to consider factors beyond interests and information, such as cooperative relationships [41], including the cooperation between local governments, the public, and businesses. In this section, the local government for the public is still used to encourage and guide the primary strategic approach, and the public for the surrounding environmental issues actively provides feedback to the local government, thus forming the interaction between the local government and the public. This section describes the local government’s green regulation of enterprises, including environmental supervision and environmental monitoring. It also emphasizes how local governments can help existing enterprises to carry out energy saving and emission reduction as well as green transformation and upgrading.

- Environmental supervision aims to check whether pollutants in industrial enterprises meet emission standards. The Industry and Information Technology Department and the Commerce Department said “we would regularly go to the enterprise to supervise and enforce the law with the ecological environment department every year. When problems are found will the enterprise be subject to law enforcement, which is mainly the responsibility of the ecological environment department”. According to one elected official, “*At present, our region has designated primary and secondary protection areas, which have different emission standards according to different protected areas. Second, we should inspect the breeding base and carry out the standardized quality transformation of breeding production facilities*” (Bureau of Agriculture and Rural Affairs, P25). Therefore, it is not difficult to see that although the ecological environment department is indispensable in environmental supervision, the active cooperation of other departments is the key to the effective implementation of green development supervision policies.
- Environmental monitoring reflects the importance of technical support and information sharing between departments. One interviewee stated that “*the department has special dynamic real-time monitoring. Whenever there is information about environmental pollution, the software is automatically updated every two hours. The data is automatically sent to the local government department*” (Bureau of Ecology and Environment, P31). Technological innovation has enabled real-time information sharing between sectors and rapidly facilitated the relevant local government departments dealing with environmental pollution problems. The Water Authority interviewees also indicated that “*the core industrial enterprises are already using the Water Resources Information Management System, which allows the Water Authority to monitor their water consumption online*” (Water Authority, P44). In addition, monitoring and testing the farming environment or waters cannot be overlooked.
- This section focuses primarily on how local governments use support policies to encourage the green transformation and upgrading of businesses. The first component is financial support. The Bureau of Industry and Information Technology and the Development and Reform Commission have established specialized funds for energy conservation and circular economy. Enterprises can obtain certain financial support if they voluntarily audit cleaner production. Simultaneously, the Bureau of Ecology and Environment and Science and Technology established a unique program to assist businesses with green development. For example, the “*Special ecological plan*” (Bureau of Science and Technology, P6) improves entrepreneurs’ understanding of green development and production technology through education and talent development policies. Local government departments also organize entrepreneurs to conduct green development education and training. Moreover, the interview participants identified that, in addition to market orientation, regulatory policies would also force enterprises to make transformations and upgrades (Bureau of Industry and Information Technology, P5).
- Energy conservation and emission reduction play a crucial role in promoting the green transformation of businesses. To enable local enterprises to produce more efficiently and environmentally friendly, local governments usually guide enterprises to carry out energy-saving technological changes to reduce energy consumption. For instance, “*focusing on the fields of energy-saving equipment, environmental engineering, green building, environmental protection services, local governments promote the cooperation of production, learning, and research of key technologies and components, and encourage the development of energy-saving and environmental protection equipment, resource recycling equipment and derivative services*” (Development and Reform Commission, P37).

An industry and information technology interviewee illustrated this point with the following example: “*Iron and steel companies have high energy consumption and high pollution. Even though they have undergone ultralow emission corrections, their energy consumption is still high, and their annual power and coal consumption are still substantial. Moreover, these businesses with high energy consumption are relatively large, making relocation difficult. We can promote enterprises’ green development through energy conservation and emission reduction. For instance, ultralow emissions, including coal consumption reduction and other factors*” (Bureau of Industry and Information Technology, P36).

Moreover, the green transformation and upgrading of diverse industries differ and are broadly divided into two categories: (1) enterprise voluntary green change and upgrading due to market-oriented industry competition and (2) the government’s strict and mandatory policies forcing enterprises to carry out green transformation and upgrading. Through data analysis, local governments adopt market-oriented standards to promote green change and upgrade enterprises. However, for heavily polluting enterprises such as electric power enterprises and chemical enterprises, local governments still draw up strict environmental standards, such as energy saving and emission reduction, to reduce the rate of environmental pollution and promote the green transformation and upgrading of enterprises.

#### 4.2.3. End-of-Pipe Green Governance

In the end, green treatment is the main guarantee for effectively implementing green development behaviors by local governments. The main emphasis in this section is on cooperative sectoral governance, the treatment of pollutants, and the strict enforcement of penalties by local governments. Notably, a growing number of researchers have argued the need to address sustainable development across policy sectors [40]. Many departments have indicated that the river chief system is a typical case of cross-sectoral cooperation from the perspective of water environment governance. Although the system still suffers from cross-functional or overlapping functions, it is undeniable that the river chief system has facilitated cross-sectoral cooperation in water environment management and has organized, coordinated, planned, coordinated, inspected, supervised, and assessed the relevant departments. The following informants elaborated on this notion: “*The river chief system includes municipal river chiefs, district river chiefs, village river chiefs, and even river section chiefs. Their responsibility is to inspect the river, and when they discover pollution issues, they will report through the river chief’s office and forward it to the appropriate department for resolution. For instance, the department of city administration is responsible for waste disposal. A disorderly discharge from a discharge pipe would be reported to the departments of Ecology and Environment and Housing and Urban-Rural Development*” (Water Authority, P45). In addition, “*there is a requirement for the river management system that to ensure the long-term maintenance of the river; it should be handed over to the corresponding river management unit to take care of the river*” (Bureau of Housing and Urban-Rural Development, P46).

- First, the end-implementing department deals with pollution, for example, “*the city administration department has some pressure as an end-implementing department and is responsible not only for the collection of waste but also for the environmentally sound treatment of waste*” (Bureau of City Administration, P28). In addition, the department has prepared a plan to construct end-of-pipe disposal facilities for the resource station of construction waste (Bureau of City Administration, P7). The second is the enforcement of penalties for violations. For example, “*polluters who exceed the emission limits are punished with fines*” (Bureau of Industry and Information Technology, P5). In addition, the most severe punitive measures are stopping production, followed by the suspension of production and rectification, some means of limiting production, administrative penalties, and a deadline for correction, which are some administrative means given by law, including two types of administrative penalties and administrative orders. Sometimes, the local government will adopt these auxiliary means, such as temporary equipment sealing.

## 5. Discussion

Through the above explanations, this study identifies this core code, namely the green development behaviors of local governments. The green development behavior of local governments mainly consists of six subcategories. Three subcategories elaborate on the formulation of local government green development policies. The green development behavior of local governments is characterized by normative, incentive, and regulatory features. The other three subcategories illustrate in detail the key processes and specific practices of local governments in implementing green development behaviors. Therefore, based on the above results, this paper constructs a theoretical model of the green development behavior of local governments, as shown in Figure 1.

A growing number of scholars recognized that local governments influence many key emission sectors, including buildings, energy supply, transportation, planning, and waste management [21]. Meanwhile, local governments play the role of facilitators and leaders in guiding the sustainable transformation of business and society [36]. However, the change in local governments to green development behaviors can be challenging [21] because the practical processes of this transformation are unknowable. Local governments are usually portrayed as institutional-level actors that play essential roles in different stages of the shift to green development behaviors [65]. Therefore, this study constructs a process framework based on new institutionalism theory and organizational process research to illustrate how organizations successfully transform into green development behaviors by formulating and implementing green development policies for different audiences emerging at different stages.

Our research categorizes local governments’ green development behaviors as policy formulation and implementation. Page mentioned that governance requires local governments to act in terms of policy authorization and implementation [41]. Rational choice institutionalism demonstrated that as implementers of green development policies implemented by the central government [40], local governments usually seek to maximize their utility. This study found that following the green development policies proposed by the central government, local governments would formulate green development plans, goals, and policies that meet their requirements based on local circumstances, and these policies usually exceed the minimum required environmental goals set by superior authorities [20]. Furthermore, sociological institutionalism highlights functions, including problem-solving processes, capacity building, and relationships [66]. Supervisors typically act in ways they believe are appropriate for their role and particular policy sector [40]. Our research reveals that organizations collaborate across sectors in implementing local government environmental regulatory policies. For example, in environmental regulation, the Department of Ecology and Environment, the Department of Water Affairs, and the Department of Housing and Urban-Rural Development actively collaborate across sectors.

This research contributes to the field by integrating the new institutionalism theory and the organizational process research to conceptualize local government green development behaviors involving multiple actors and to articulate critical processes and practices. New institutionalism theory and organizational process research could collectively explain organizational change [12,31,32,33]. This evolutionary perspective explains how organizational practice processes emerge, unfold, and end over time [45]. This study identifies local government green development behavior as a behavioral transformation, meaning the behavior taken by local governments to change from the original development model that only pursues economic growth to a green development model that seeks environmental protection and economic growth. Its purpose is to encourage enterprises to realize the greening of their production methods and encourage residents to learn the greening of their lifestyles. The components of local governments’ green development behaviors are explained, and the key processes and practices of such behaviors are analyzed, which help to spread green development behaviors among different local government departments.

Previous research has described the mediating role of local governments in facilitating partnerships between sustainable development goals and the private sector [1]. Our analysis confirms the facilitating role of local governments in greening businesses. Furthermore, our study complements how local governments can facilitate the public’s realization of green living and promote green production in different enterprises. Additionally, our findings suggest that different periods and sectors of the organizational process are critical for implementing green development practices [33]. As shown in Figure 1, during the source control stage of the project, guidance and incentive policies are implemented for the public, while more mandatory policies are added for the enterprises. Local governments must fulfill green regulations such as environmental assessment when introducing new businesses. As the process evolved, the second stage dealt with green rules for the public. Although it was still dominated by the implementation of incentives and guidance policies, it was found that the public would interact by providing feedback to the local government authorities. Currently, local governments are more inclined to regulate the enterprises already in the region and promote green transformation and upgrading. Mandatory policies dominate the final stage of end-to-end green governance. While incentive and guidance policies are valuable in the first two stages, the continued use of these practices in the later stage can prevent local governments from implementing green development behaviors. Therefore, we describe local governments’ green development behaviors as an evolutionary process, the outcome of which depends on the sequence and speed of different practices [33,44] and on different local government departments. Moreover, it is worth noting that local governments in China pay more attention to enterprises’ green production than the public’s green life. However, existing studies set in Europe [21,34] and the United States [18] emphasize the relationship between communities, residents, and local governments’ sustainable practices [35]. However, local governments and private sector partnerships cannot be ignored [1].

Overall, most of our research focused on local government departments that are closely associated with green development. By introducing the concept of green development, the effective implementation of policies related to green development and the realization of regional green development has become an ongoing process for many local government departments. This is due to the ever-changing institutional environment in which local governments operate and the growing number of stakeholders actively engaged in regional green development. The green development of enterprise production modes and public lifestyles urgently requires detailed guidance from local governments. Therefore, understanding the key processes and practices of local governments’ green development behaviors facilitates the clarification of responsibilities and cross-sectoral collaboration among local government departments and contributes to a better comprehension of green development by businesses, the public, and other stakeholders. Furthermore, feedback from enterprises and the public on the implementation of green development policies contributes to the further improvement of green development policy formulation by local governments, thus enhancing the effectiveness of policy implementation.

## 6. Conclusions and Implications

### 6.1. Conclusions

This study analyzed local governments’ green development behaviors from an organizational perspective, combining new institutionalism and organizational process research. By conducting in-depth interviews with 53 local government officials from nine government departments related to green development and analyzing case studies of local governments in China, we found that the green development behavior of local governments mainly consists of the formulation and implementation of local government green development policies. The formulation of local government green development policies contains three subcategories, reflecting the normative, incentive, and regulatory characteristics of local governments’ green development behaviors. Local governments’ key processes and specific practices in implementing green development behaviors include project source control, process green monitoring, and end green treatment.

### 6.2. Implications

This study provides new perspectives for local governments to formulate localized green development policies and study the effective implementation of green development behaviors. Additionally, this study proposes the following recommendations: First, the central government should establish new incentive mechanisms and long-term performance evaluation systems for local governments to prompt them to transfer their focus from current short-term economic goals to long-term green development. Second, for enterprises, local governments must continuously update new measures to measure the results and effectiveness of implementing industry standards in actual projects. Assessment methods, such as environmental management systems, green production, and carbon audits, can potentially improve the behavior of industries towards green and low-carbon development. In addition, local governments should further promote the greening of public lifestyles and encourage the public to purchase green products. Local governments should also adopt more incentives for the waste separation and recycling industry to reduce landfills and increase the resource recovery rate.

Limitations are that this study focused on three cities in Jiangsu Province as the case studies and centered on sectors related to green development. Future research could represent how individuals in different organizations or sectors perceive green development behaviors. Additionally, this study found that local government departments show inconsistent concern for the public’s green lifestyles and the enterprises’ green production, and future research necessitates the strengthening of in-depth studies on green lifestyles. Moreover, future research would be necessary to understand the influence of stakeholders or other factors on implementing green development behaviors, which would help local governments to further realize regional green development.

## Figures and Tables

**Table 1:** Research related to green development of local governments in China.

References	Research Method	General Findings
Zhou et al. (2023) [24]	Data from 30 provinces in mainland China from 2001 to 2020, empirical analysis.	Regional economic development is related to both the internal and external environment and the characteristic behavior of local governments.
Xin et al. (2022) [25]	Data on fiscal decentralization, land resource use, and pollutant emissions in China, 2011–2018.	Local government leaders tend to prioritize short-term economic growth over environmental protection when they are under intense pressure for promotion.
Liu et al. (2022) [26]	Provincial panel data from 2008 to 2018, spatial econometric approach.	Local governments create a peer effect in green governance activities.
Li et al. (2022) [3]	18 literatures, meta-analysis.	Enterprise economic behavior, environmental behavior, social behavior, and public participation are significantly and positively influenced by the government’s green development behavior.
Chen & Gao (2022) [29]	Evolutionary game.	Urban residents are more likely to change their green decisions under positive government incentives, but firms’ behavioral decisions are mainly influenced by government penalties.
Liu et al. (2021) [15]	Dynamic evolutionary game models.	Exploring group behavior of local governments in green governance from a knowledge management perspective.
Liu et al. (2021) [30]	308 civil servants working in the public sector, empirical study.	Green behavioral intentions, organizational environmental strategies, and green lifestyles have a positive influence on green behaviors.
Peng (2020) [27]	Panel data for 274 Prefecture-Level Cities in China, 2005–2015, Composite Indicators of Environmental Regulation	Interregional interaction of environmental regulatory strategies influences green productivity.
Wu et al. (2020) [37]	Empirical analysis of sample data from 30 provinces in China.	Environmental legislation and regulation can improve regional ecological quality.
Liu et al. (2018) [38]	Multi-adaptive scenario system dynamics modeling.	Five key policy variables were identified, including urban population carrying capacity, water consumption and recycling rates, and expansion of urban land cover.
Huang et al. (2017) [17]	Questionnaires and semi-structured interviews.	The density of the network ranges from weak to moderate, leading to collective action problems and insufficient cooperative governance.
Chen et al. (2016) [28]	Job changes of 31 governors between 1978 and 2012, probit model empirical testing.	Energy productivity has a significant positive effect on the political promotion of Chinese governors.

**Table 2:** Descriptions of sample information.

Characteristics	City A	City B	City C
Location within province	South-East	South	Central
Population	7.49 million	5.37 million	3.22 million
Interviews with divisional leaders	4	3	8
Interviews with section leaders	10	12	16
Archival data (total number of documents)	4	9	10
Interview time in the local sector (hours)	Ranging in duration from 0.5 to 1.5 h for each interview		

**Table 3:** Interviewee’s characteristics.

Characteristics	Data Categories	No. of Participants	Percentage
Gender	Male	40	75.47%
	Female	13	24.53%
Age	21~30 years old	6	11.32%
	31~40 years old	18	33.96%
	41~50 years old	22	41.51%
	51~60 years old	7	13.21%
Level of education	Associate degree	3	5.66%
	Bachelor’s degree	24	45.28%
	Master’s degree	20	37.74%
	Doctoral degree	6	11.32%
Department	Development and Reform Commission	8	15.09%
	Bureau of Industry and Information Technology	5	9.43%
	Bureau of Ecology and Environment	5	9.43%
	Bureau of Science and Technology	5	9.43%
	Bureau of City Administration	7	13.21%
	Bureau of Agriculture and Rural Affairs	5	9.43%
	Bureau of Commerce	4	7.55%
	Water Authority	8	15.09%
	Bureau of Housing and Urban-Rural Development	6	11.32%

**Table 4:** Key processes and practices of green development behavior of local governments.

Empirical Themes	Conceptual Categories	Categories
Statements describing “green industry guidance catalog”, “emerging industry strategy catalog”, and “green industry policy”	Guiding industrial green production policy	Green development guiding policy formulation
Statements describing “green lifestyle”, “subsidy policy for purchasing green products”, “green travel policy”, and “green prevention and control technology of crop diseases”	Guiding public green living policy	
Statements describing “special funds for circular economy”, “special funds for cleaner production”, “bank financing policies”, and “tax preferential policies”	Financial incentive policy	Green development supporting policy formulation
Statements describing “cleaner production training for business managers”, “environmental policy explanation”, “green technology guidance”, and “technology support”	Education and talent support policy	
Statements describing “employee re-employment”, “personnel placement”, and “financial compensation”	Social security policy	
Statements describing “water saving and pollution reduction”, “rainwater recycling”, “energy-saving technology transformation”, “water saving”, material saving, land saving, and energy saving. Environmental protection mainly refers to indoor environmental protection	Energy saving and emission reduction policy	Green development regulatory policy formulation
Statements describing “new environmental protection law”, “industry regulatory standards”, “environmental evaluation standards”, “corporate social environment credit”, “operation management standards”, “environmental taxes”, “environmental resource prices”, and “emissions trading”	Environmental regulatory policy	
Statements describing “local government—accountability”, “enterprise—environmental damage compensation”, “production shutdown”, “production restrictions”, and “administrative penalties”	Enforcement and punishment policy	
Statements describing how local governments “promote green concepts”, “publicize green ecological aquaculture models”, and “advocate green buildings”	Publicizing green development policies	Project source control
Statements describing how local governments “guide enterprises to voluntary green production” and “encourage residents to buy green products”	Guiding and supporting green development policy implementation	
Statements describing how local governments provide “certain plant rent concessions”, “incentive policies in the stages of enterprise incubation and industrial transformation”, and “financial credit”	Implementation of supporting policies for newly introduced enterprises	
Statements describing how local governments “implement green industry guidance catalog”, “emerging industry strategy catalog”, “green industry policy”, and “environmental evaluation standards”	Enterprise access standards	
Statements describing how local governments implement “environmental supervision” and “law enforcement”	Environmental supervision	Process green regulation
Statements describing how local governments dynamically detect “wastewater” and “exhaust gas” from enterprises or residents	Environmental monitoring	
Statements describing how local governments provide supporting policies for the existing enterprises, such as “specialized funds for energy conservation and circular economy” and “special ecological plan”	Implementation of supporting policies for existing enterprises	
Statements describing how local governments provide “energy-saving technological to reduce energy consumption ”, “ultra-low emissions, including coal consumption reduction”, and encourage “the development of energy-saving and environmental protection equipment, resource recycling equipment and derivative services”, “enterprise voluntary green change and upgrading”, and “force enterprises to carry out green transformation and upgrading”	Energy conservation and emission reduction and green transformation and upgrading	
Statements describing how local governments implement “end-of-pipe treatment”, such as “water environment treatment” and “harmless waste treatment”	Pollution treatment	End-of-pipe green governance
Statements describing how local governments implement “administrative penalties” and “administrative orders”.	Enforcement and punishment	

## Data Availability

The datasets used and/or analyzed during this current study are available from the corresponding author upon reasonable request. Please contact the corresponding author for the data requests.
